# Research on the income distribution effect of factor allocation efficiency changes among China’s three major industries

**DOI:** 10.1371/journal.pone.0292927

**Published:** 2023-10-26

**Authors:** Bochao Zhang, Wanhao Dong, Jin Yao

**Affiliations:** 1 Institute of Economics, Shanghai Academy of Social Sciences, Shanghai, China; 2 School of Public Finance and Administration, Shanghai Lixin University of Accounting and Finance, Shanghai, China; East China Normal University, CHINA

## Abstract

This paper aims to study the impact of correcting the factor misallocation among China’s three major industries on China’s income gap and income distribution pattern. By using the industry Panel data at the provincial level in China, we measure the degree of factor misallocation among the three major industries in China’s provinces from 2002 to 2019 by building a factor misallocation measurement model, and then uses reverse thinking to compare the income gap under the condition of no factor misallocation with the actual income gap, and then obtains the impact of factor misallocation on the income gap, And use this method to focus on analyzing the impact of factor allocation efficiency changes among the three industries on income distribution pattern. The research finds that: (1) There is a serious factor misallocation among the three major industries in each province. From the perspective of subdivided factors, the factor misallocation among the three major industries in China’s provinces is mainly caused by labor misallocation. Factor misallocation shows a trend of convergence first and then divergence among regions. (2) There is a strong heterogeneity in the explanatory power of different dimensions of the income gap of factor misallocation among the three major industries in China’s provinces. Among them, correcting the misallocation of total factors among the three major industries in China’s provinces can only narrow the internal income gap of the tertiary sector of the economy, and expand the internal income gap between the primary and secondary industries. (3) The impact of correcting the total misallocation, capital misallocation and labor misallocation among the three industries on the income gap among industries or provinces is narrowing first and then expanding. (4) Further research shows that although the level of factor misallocation among provinces in China is significantly lower than the average level of factor misallocation among the three major industries within each province, it has a stronger explanatory power for the inter-provincial income gap. Correcting the total factor misallocation and labor misallocation among provinces can significantly reduce the inter-provincial income gap in China. Correcting the total factor misallocation and labor misallocation among provinces in 2019 can reduce the inter-provincial income gap by 51.48% and 81.68% respectively. Only correcting the capital misallocation among provinces will expand the inter-provincial income gap, and only correcting the capital misallocation among provinces in 2019 will expand the inter-provincial income gap by 112.21%.

## Introduction

The Fifth Plenary Session of the 19th Central Committee of the Communist Party of China stressed the need to "solidly promote common prosperity" and proposed that by 2035, "more obvious and substantial progress in the common prosperity of all the people will be achieved". General Secretary Xi Jinping stressed again at the 27th collective study of the Politburo of the CPC Central Committee: "To enter a new stage of development and implement the new development concept in a complete, accurate and comprehensive manner, we must pay more attention to the issue of common prosperity." In the context of the new stage of development, on the one hand, the focus of China’s economic and social development will gradually shift from attaching importance to the "high growth rate" of economic scale to improving efficiency and quality, that is, it is necessary to change from the extensive factor input-based high-speed economic growth model in the past to a total factor productivity-driven economy [1]. On the other hand, the realization of the goal of common prosperity requires that the income gap between residents be prevented from becoming too large. As far as the former is concerned, one of the important ways to improve the overall total factor productivity of the economy is to correct the structural misallocation of factors and improve the efficiency of factor structural allocation. According to previous research literature, the improvement of China’s total factor productivity in the future will increasingly depend on the improvement of factor structural allocation efficiency [2]. In the latter case, the re-flow allocation of factors between different sectors makes the factors flow smoothly from low-efficiency sectors to high-efficiency sectors, thereby changing the marginal output and marginal benefits of capital and labor production factors, which is inevitable to have a significant impact on income disparities within and among different sectors. The flow of factors of production between different industries will change the Marginal cost of labor in different industries, which corresponds to the changes in the wage distribution structure and average wage of labor in different industries, thus affecting the income gap within and among industries [3].This raises a question worth paying attention to, what impact will correcting the misallocation of structural factors in different dimensions of China’s economy have on China’s income distribution pattern while improving China’s total factor productivity? Can correcting the misallocation of structural factors in different dimensions achieve the win-win goal of high-quality economic development and narrowing the income gap, optimizing the income distribution pattern? In-depth research on the above issues is of great significance for China’s future further promotion of supply-side structural reform, correction of distortion in factor allocation and creation of a fair and just income distribution pattern.

Due to market segmentation, sectoral monopoly and institutional mechanisms, the misallocation of factor allocation among China’s three major industries has existed for a long time. There is surplus labor and a lack of capital investment in agriculture and rural areas. The Secondary sector of the economy and Tertiary sector of the economy are facing increasing difficulties in recruiting workers, rising labor costs and capital expanding disorderly. Therefore, it is necessary to construct a model to examine the degree of factor misallocation among China’s three major industries. Based on the perspective of the three major industries, this article constructs a model that puts labor and capital under a unified framework, and calculates the total degree of misallocation among the three major industries in various provinces of China from 2002 to 2019, as well as the degree of misallocation of labor and capital among the three major industries. At the same time, this paper focuses on analyzing the impact of factor misallocation among the three major industries on the income gap among provinces, industries, and within each of the three major industries.

The potential marginal contribution of this article is manifested in two aspects: firstly, in terms of measurement methods, based on the perspective of the three major industries, a measurement model for factor misallocation among the three major industries has been constructed under a unified framework, and the model has been simplified from the three-layer structure of Hsieh and Klenow [4] to a two-layer structure, allowing it to directly use industrial sector data to measure factor allocation efficiency, without relying on micro data at the enterprise level, The applicability of the element misallocation calculation model has been expanded, and this model lays the foundation for later research on the efficiency of element allocation. Secondly, in terms of research content, it focuses on analyzing the impact of factor misallocation among the three major industries on income disparities. Expanding the research on factor misallocation to the field of income distribution has taken a meaningful step forward in exploring the underlying mechanism of the impact of factor misallocation on economic growth.

### Literature review

According to Banerjee and Moll [5], "factor misallocation" (or "resource misallocation") has two meanings: one refers to the allocation of production factors such as labor and capital among various microeconomic agents or microeconomic sectors participating in economic activities that fail to maximize output under the condition of resource scarcity, that is, connotative factor misallocation; the other refers to the dynamic replacement of economic entities in an ineffective state, that is, inefficient subjects do not withdraw from the market or cannot exit the market, resulting in their inefficient occupation of production factors, and high-efficiency subjects cannot enter the market, which eventually leads to too much proportion of inefficient subjects in economic activities and too low proportion of high-efficiency market subjects, which is called the epitaxial factor misallocation [4]. Both of these types of factor misallocations result in losses in total factor productivity and total output. The connotative resource misallocation presents more complex multi-dimensional structural characteristics, among which industrial structure imbalance, ownership discrimination and inter-regional market segmentation are the three important structural factors leading to the distortion of China’s resource allocation. In terms of inter-industry distortion. Restuccia and Rogerson [6] summarized literature on misallocation and productivity that focus on the reallocation of factors across heterogeneous production units as an important source of measured TFP differences across countries. Yuan and Xie [7] based on the Aoki model [8] pointed out that the distortion allocation between the agricultural sector and the non-agricultural sector led to a TFP loss of 2%-18%. Chen and Hu [9] based on the Aoki model pointed out that the distortion between subindustries within China’s manufacturing industry led to a 15% loss of output. However, Han and Zheng [10] based on the HK model [4] pointed out that the distortion of factor allocation between subindustries within the manufacturing industry only leads to a TFP loss of 4.72%. In terms of inter-regional twisting, Brandt et al. [11] based on the extended HK model pointed out that the distortion of inter-provincial factor allocation caused TFP loss by 8% (which is called BTZ model for short), and Jin [12] based on the HK model and BTZ model calculated that China’s inter-provincial resource misallocation caused an average annual TFP loss of 9.71%. In terms of ownership structure distortion, Chen [13] based on the DSGE model estimated that the distortion of factor allocation between different ownership departments leads to a 19% TFP loss. Jin et al. [14] based on the HK model found that ownership discrimination leads to a TFP loss of 200% by using micro-enterprise data analysis of Chinese industrial enterprise database. Zhang and Zhang [15] based on the HK model pointed out that the distortion of factor allocation under the difference of ownership only leads to a TFP loss of 7.4%.

Numerous scholars in China have conducted in-depth analysis on the reasons for the factor misallocation in Chinese. Some scholars focus on the government system, such as exploring the impact of development strategy, registered residence system, public pension insurance system, etc. on income distribution [16–18]. Another part of scholars focus on exploring the impact of economic globalization, economic growth, industrial structure, Imperfect competition, etc. on income gap from the market level [19–22]. Some scholars have also conducted research on the change law of China’s income distribution structure [23, 24].

In summary, the impact of factor misallocation on economic growth has become an important tool to explain the development gap between regions or countries [5]. The so-called regional development gap generally refers to the significant difference in economic aggregate (GDP) or per capita GDP, and to a large extent, the difference of per capita GDP represents the income gap between different regions, that is, factor misallocation obviously has a significant income distribution effect. Existing studies on the income distribution effect of factor misallocation have begun to cover, but they have not been comprehensively in-depth. Cai [25] found that the significant differences in the degree of labor market distortion in different regions of China are one of the important reasons for the efficiency difference and income gap between regions in China. Lai [26] believed that the misallocation of production factors between urban and rural areas is an important reason for the widening of urban-rural income gap. Wu [27] also analyzed the income gap between urban and rural areas and antipoverty in rural areas, and argued that the misallocation of factors led to insufficient total social products, and at the same time, the long term existence of the urban-rural dual structure led to the inability to achieve fair distribution of total social products between urban and rural areas in the distribution process, which was an important reason for the concentration of poor people in rural areas. Yang and Bai [28] empirically examined the impact of factor misallocation in the dual economy on the income gap by constructing a binary economic model, and found that the misallocation of binary economic factors in 2010 could explain the income gap between provinces and within the agricultural sector in China by 15.7% and 25.1% respectively. Liu et al.[29] examined the impact of factor misallocation on China’s regional unbalanced development, and found that sectoral total factor productivity, factor misallocation and factor endowment could explain 56.5%, 30.7% and 12.8% of regional unbalanced development respectively. Guo and Zhang [30] found that alleviating the factor misallocation between China’s agricultural and non agricultural sectors can significantly reduce the urban-rural income gap in China. Some scholars have also examined the impact of land resource allocation efficiency on income distribution patterns. For example, Li et al [31] found that land resource misallocation can expand the income gap between urban and rural areas in China through independent intermediary channels of industrial structure and unbalanced urbanization.

In terms of factor allocation efficiency measurement methods, Aoki mainly measures the level of efficiency loss caused by factor misallocation by constructing factor allocation distortion friction coefficient, while Hsieh and Klenow [4] use the Statistical dispersion of micro enterprises’ Total factor productivity to express the level of factor misallocation. Bartelsman and Doms [32] also use the dispersion of Total factor productivity among enterprises to measure the distortion of factor allocation. Swiecki [33] used wage differentials between industries to measure the degree of factor distortion. Li et al. [34] used the direct distance function (DDF)—Data Envelopment Analysis (DEA) model to measure the green total factor productivity of laying hens in China.

Based on the above analysis, it can be found that the relevant research on the construction and quantitative evaluation of factor misallocation measurement model has been very rich, and according to the existing research results, there is a serious multi-dimensional structural element misallocation problem in China. At the same time, some studies have begun to expand the study of factor misallocation to the field of income distribution, but most of the existing studies examine the income distribution effect from the perspective of urban and rural dual economic factor misallocation, and the income distribution effect of factor misallocation between industries involves less frequently. Moreover, most of the income disparities involved in the existing studies are mainly inter-regional or urban-rural income gaps, and there is no complete comparative analysis of the impact of factor misallocation on income gaps in different dimensions (inter-industry, intra-industry, inter-region, intra-region). Therefore, on the basis of existing research, it is necessary to carry out extended research on the impact of factor misallocation on the income gap in other dimensions, so as to more comprehensively investigate and understand the income distribution effect of multi-dimensional structural factor misallocation in China.

According to Bai [35], the misallocation of factors among China’s three major industries is an important cause of the loss of total output efficiency and industrial structure imbalance in China. Correcting the misallocation of factors between the three major industries and promoting the rational flow and efficient allocation of labor and capital factors among the three major industries are not only an inevitable requirement for promoting the transformation and upgrading of China’s industrial structure, but also of great significance for building a modern industrial system, improving the quality and efficiency of the supply system, and even effectively promoting high-quality economic development. Therefore, based on the perspective of the three major industries, this paper quantitatively evaluates the level of factor misallocation between the three major industries in various provinces of China and analyzes its main characteristics by constructing a factor misallocation model. Then, this paper focuses on the impact of the misallocation of the three major industries on the income gap between provinces, within industries and between the three major industries. In addition, considering that China is accelerating the construction of a unified domestic market, this requires that production factors not only improve the misallocation of various structural factors within each province, but also free flow and efficient allocation between provinces in a larger spatial range have become inevitable requirements and general trends in the future. Therefore, this paper urgently needs to use this research framework to further analyze the impact of China’s inter-provincial factor misallocation between China’s provinces on the income gap between China’s provinces, so as to fully compare the impact of structural factor misallocation in the two spatial dimensions of provincial and inter-provincial on the income distribution pattern in China, and provide theoretical and empirical support for China’s next promotion of supply-side structural reform, construction of fair and just income distribution pattern and construction of a unified domestic market.

### Model construction

#### Construction of factor misallocation measurement model between three major industries

Previous models for measuring factor misallocation mostly followed Aoki’s model construction method [8], using the degree of output loss caused by misallocation to represent the degree of factor misallocation. The final calculation formula is:

$$\begin{array}{l} (Y/Y_{efficient})=\prod_{i=1}{\left(\frac{{\left(\frac{s_{it}\beta_{Ki}}{\beta_{K}}\gamma_{Kit}K_{t}\right)}^{\beta_{Ki}}{\left(\frac{s_{it}\beta_{Li}}{\beta_{L}}\gamma_{Lit}L_{t}\right)}^{\beta_{Li}}{\left(\frac{s_{it}\beta_{Mi}}{\beta_{M}}\gamma_{Mit}M_{t}\right)}^{\beta_{Mi}}}{{\left(\frac{s_{it}\beta_{Ki}}{\beta_{K}}K_{t}\right)}^{\beta_{Ki}}{\left(\frac{s_{it}\beta_{Li}}{\beta_{L}}L_{t}\right)}^{\beta_{Li}}{\left(\frac{s_{it}\beta_{Mi}}{\beta_{M}}M_{t}\right)}^{\beta_{Mi}}}\right)}^{s_{it}}\\ \;=\prod_{i=1}{\left({\left(\gamma_{Kit}\right)}^{\beta_{Ki}}{\left(\gamma_{Lit}\right)}^{\beta_{Li}}{\left(\gamma_{MAt}\right)}^{\beta_{MS}}\right)}^{s_{it}} \end{array}$$

Among them, *i* represents the industry, *s*_*i*_ represents the industry share, and *K*, *L*, *M*, and *Y* represent capital, labor, intermediate input, and output, respectively. γ Represents the relative distortion coefficient of the factor, $\gamma_{Ki}=(\frac{K_{i}}{K})/(\frac{s_{i}\beta_{Ki}}{\beta_{K}})$, $\gamma_{Li}=(\frac{L_{i}}{L})/(\frac{s_{i}\beta_{Li}}{\beta_{L}})$, $\gamma_{Mi}=(\frac{K_{M}}{K})/(\frac{s_{i}\beta_{Mi}}{\beta_{K}})$, However, we can see that in the first step of the above formula, the output share *s*_*i*_ of the *i* industry is obtained in the case of misallocation, while the *s*_*i*_ in the denominator is the output share in the absence of misallocation. The meanings and values represented by the two are not the same, so they cannot be mutually reduced to obtain the expression in the second step. However, the output share *s*_i_^*^ in the effective state cannot be calculated through a consistent formula within the model. In view of this, this paper refers to the model construction idea of Brandt et al. [11], assuming that the economy is composed of three major industrial sectors (primary industry, secondary industry and tertiary-industry, the same below), and the output of the three industrial sectors cooperates with each other to obtain the total output Y, that is, Y is the CES production function of Yi (the annual output of industry i), expressed as follows: $Y=\sum_{i=1}^{N}(\theta_{i}Y_{i}^{\sigma }{)}^{1/\sigma }$, where $\sum_{i=1}^{N}\theta_{i}=1$ and *θ*_*i*_ represents the weight of the output of industry i in the total output production process. Continuing to assume that the production function of each industrial sector is $Y_{i}=A_{i}K_{i}^{\alpha }L_{i}^{1-\alpha }$, and there is $K=\sum_{i=1}^{N}K_{i},L=\sum_{i=1}^{N}L_{i}$, the ratio of capital and labor input in the three industrial sectors: $k_{i}=\frac{K_{i}}{K},l_{i}=\frac{L_{i}}{L}$.

This gives us the formula for calculating the overall total factor productivity of the economy:

$$TFP=(\sum_{i=1}^{N}\theta_{i}Y_{i}^{\sigma }{)}^{1/\sigma }/(K^{\alpha }L^{1-\alpha })=[\sum_{i=1}^{N}\theta_{i}(A_{i}k_{i}^{\alpha }l_{i}^{1-\alpha }{)}^{\sigma }{]}^{1/\sigma }$$ (1)

It is further assumed that the prices of industrial capital and labor factors are $\tau_{i}^{k}r$ and $\tau_{i}^{l}w$, where $\tau_{i}^{k}r$ and $\tau_{i}^{l}w$ represent the price distortion coefficient of capital and labor respectively. Then the profit maximization problem of total output is: $\underset{Y_{i}}{max}\{P(\sum_{i=1}^{N}\theta_{i}Y_{i}^{\sigma }{)}^{1/\sigma }-\sum_{i=1}^{N}P_{i}Y_{i}\}$, and the profit maximization problem of output of industrial sectors is: $\underset{K_{i},L_{i}}{max}\{P_{i}A_{i}K_{i}^{\alpha }L_{i}^{1-\alpha }-\tau_{i}^{k}rK_{i}-\tau_{i}^{l}wL_{i}\}$. The proportion of capital input and labor input in each industrial sector in a distorted state are: $k_{i}=\frac{K_{i}}{K},\;l_{i}=\frac{L_{i}}{L}$:

$$k_{i}=\frac{\theta_{i}^{\frac{1}{1-\sigma }}{{\bar{A}}_{i}}^{\frac{\sigma }{1-\sigma }}\tau_{i}^{k-1}}{\sum_{i=1}^{N}\theta_{i}^{\frac{1}{1-\sigma }}{{\bar{A}}_{i}}^{\frac{\sigma }{1-\sigma }}\tau_{i}^{k-1}},\;l_{i}=\frac{\theta_{i}^{\frac{1}{1-\sigma }}{{\bar{A}}_{i}}^{\frac{\sigma }{1-\sigma }}\tau_{i}^{l-1}}{\sum_{i=1}^{N}\theta_{i}^{\frac{1}{1-\sigma }}{{\bar{A}}_{i}}^{\frac{\sigma }{1-\sigma }}\tau_{i}^{l-1}}$$ (2)

Thereinto, $\bar{A_{i}}=A_{i}{\left(\tau_{i}^{k}\right)}^{-\alpha }{\left(\tau_{i}^{l}\right)}^{\alpha -1}$. Substituting the expression (2) of the proportion of capital and labor input in each industrial sector into Eq (1), the overall total factor productivity of the economy in a distorted state is:

$$TFP=(\sum_{i=1}^{n}\theta_{i}^{\frac{1}{1-\sigma }}{\bar{A_{i}}}^{\frac{\sigma }{1-\sigma }}{)}^{\frac{1-\sigma }{\sigma }}\times (\frac{\sum_{i=1}^{n}\theta_{i}^{\frac{1}{1-\sigma }}{\bar{A_{i}}}^{\frac{\sigma }{1-\sigma }}\tau_{i}^{K-1}}{\sum_{i=1}^{n}\theta_{i}^{\frac{1}{1-\sigma }}{\bar{A_{i}}}^{\frac{\sigma }{1-\sigma }}}{)}^{-\alpha }\times (\frac{\sum_{i=1}^{n}\theta_{i}^{\frac{1}{1-\sigma }}{\bar{A_{i}}}^{\frac{\sigma }{1-\sigma }}\tau_{i}^{L-1}}{\sum_{i=1}^{n}\theta_{i}^{\frac{1}{1-\sigma }}{\bar{A_{i}}}^{\frac{\sigma }{1-\sigma }}}{)}^{\alpha -1}$$ (3)

Therefore, it can be obtained that when there is no misallocation of resources between the three major industrial sectors, that is, in the effective state ($\tau_{i}^{k}=\tau_{i}^{l}=1$), the effective capital investment ratio $k_{i}^{*}$ and effective labor investment ratio $l_{i}^{*}$ of each industrial sector:

$$k_{i}^{*}=\frac{\theta_{i}^{\frac{1}{1-\sigma }}{A_{i}}^{\frac{\sigma }{1-\sigma }}}{\sum_{i=1}^{N}\theta_{i}^{\frac{1}{1-\sigma }}{A_{i}}^{\frac{\sigma }{1-\sigma }}},l_{i}^{*}=\frac{\theta_{i}^{\frac{1}{1-\sigma }}{A_{i}}^{\frac{\sigma }{1-\sigma }}}{\sum_{i=1}^{N}\theta_{i}^{\frac{1}{1-\sigma }}{A_{i}}^{\frac{\sigma }{1-\sigma }}}$$ (4)

Substituting Eq (4) into Eq (1), the overall total factor productivity of the economy in the effective state is obtained:

$$TFP^{*}={\left(\sum_{i=1}^{N}\theta_{i}^{\frac{1}{1-\sigma }}A_{i}^{\frac{\sigma }{1-\sigma }}\right)}^{\frac{1-\sigma }{\sigma }}$$ (5)

As mentioned above, this paper expresses the degree of resource misallocation caused by differences between the three major industrial sectors in terms of total factor productivity losses. The degree of total factor productivity loss caused by the total factor misallocation between the three major industrial sectors can be obtained:

$$d=\frac{TFP^{*}}{TFP}-1$$ (6)

This paper continues to make the labor price distortion coefficient $\tau_{i}^{l}$ and the capital price distortion coefficient $\tau_{i}^{k}$ equal to 1, and then examine the degree of total factor productivity loss caused by capital misallocation and labor misallocation respectively. The first-order condition for profit maximization in the industrial sector is obtained: $\tau_{i}^{K}\propto \frac{Y_{i}^{nor}}{K_{i}},\tau_{i}^{L}\propto \frac{Y_{i}^{nor}}{L_{i}}$. According to the first-order condition of profit maximization of overall output, the formula of calculating *θ*_*i*_ is obtained is $\theta_{i}=\frac{1}{T}\sum_{t=1}^{T}\frac{P_{i}(t){\left[Y_{i}^{nor}(t)/P_{i}(t)\right]}^{\sigma }}{\sum_{i=1}^{N}P_{i}(t){\left[Y_{i}^{nor}(t)/P_{i}(t)\right]}^{\sigma }}$. This paper continues to refer to the method of Brandt and Zhu [36] for capital-output elasticity α = 0.45, and refer to the method of Brandt et al.[11] for σ = 1/3. We sort the above data indicators into Panel data, and then use stata software to calculate according to the above steps. The factor allocation efficiency calculation model constructed in this article can effectively avoid the aforementioned shortcomings of existing traditional models.

#### Construction of measurement model for the impact of factor misallocation on the income distribution pattern among three major industries

In this paper, the coefficient of variation of income distribution is mainly selected as a proxy indicator to measure income inequality. Based on the research methods of Caselli [37] and Bai and Yang [38], it is no longer used for before-and-after comparisons using variance in per capita income, but the coefficient of variation of per capita income after removing factor distortion is compared with the coefficient of variation of real per capita income. This treatment can effectively avoid the interference of dimensional differences, and the specific expression of S is as follows:

$$S=\frac{CV(y^{*})}{CV(y)}$$ (7)

Among them, the actual per capita income within the three major industries in each province is yi = $\frac{Y_{i}}{L_{i}}=A_{i}K_{i}^{\alpha }L_{i}^{-\alpha }$, Actual per capita income of the three major industries in each province after eliminating distortion in labor factor allocation is $y_{i}^{*}=A_{i}K_{i}^{\alpha }{\left(l_{i}^{*}L\right)}^{-\alpha }$, Actual per capita income of the three major industries in each province after eliminating distortion in capital factor allocation_is $y_{i}^{*}=A_{i}L_{i}^{-\alpha }{\left(k_{i}^{*}K\right)}^{\alpha }$, Actual per capita income of the three major industries in each province after simultaneously eliminating distortions in the allocation of capital and labor factor is $y_{i}^{*}=A_{i}{\left(l_{i}^{*}L\right)}^{-\alpha }{\left(k_{i}^{*}K\right)}^{\alpha }$. *CV*(y*) is the coefficient of variation of per capita income after eliminating the misallocation of factors (capital, labor, capital and labor) among the three major industries. *CV*(y*) is the coefficient of variation of per capita income after eliminating the distorted allocation of factors between the three major industries, *CV*(y) is the coefficient of variation of per capita income when there is a distorted allocation of factors between the three major industries in the actual state. S represents the remaining income gap caused by other factors after eliminating the distortion allocation of factors between the three major industries, then 1-S can be understood as the impact of the distortion allocation of factors between the three major industries on the income gap, the smaller the S, indicating that the more the distorted allocation of factors between the three major industries explains the income gap.

The income gap between the three major industries is expressed by the between-group variance formula, and the per capita income of the three major industries in each province is divided into three groups, and the income gap between the three major industries in each province is calculated according to the between-group variance formula:

$$\sigma_{I}=\sum_{i=1}^{m}f_{i}{\left(\bar{x_{i}}-\bar{x}\right)}^{2}/\sum_{i=1}^{m}f_{i}$$ (8)

Among them, $\bar{x_{i}}$ is the average per capita income of each province in the respective groups of the three major industries, $\bar{x}$ is the average per capita income of each province as a whole, and *f*_*i*_ is the frequency of each group. The impact of the factor misallocation on the income gap between the three major industries expressed by the variance between industries is as follows:

$$S^{\prime }=\frac{\sum_{i=1}^{m}f_{i}{\left({\bar{x_{i}}}^{*}-{\bar{x}}^{*}\right)}^{2}/\sum_{i=1}^{m}f_{i}}{\sum_{i=1}^{m}f_{i}{\left(\bar{x_{i}}-\bar{x}\right)}^{2}/\sum_{i=1}^{m}f_{i}}$$ (9)

In the Formula (9), ${\bar{x_{i}}}^{*}$ and ${\bar{x}}^{*}$ are the average per capita income of each province in the three major industrial groups and the average per capita income of each province as a whole after correcting the misallocation of factors between the three major industries respectively. We sort the above data indicators into Panel data, and then use excel software to calculate according to the above steps.

### Data processing and source description

In order to measure the level of factor misallocation between the three major industries in China’s provinces from 2002 to 2019 and its impact on income disparity, it is necessary to need the annual added value, capital stock and labor force data of the three major industries in each province in China, and add up the data of the three major industries in the above provinces to obtain the annual data on added value, capital stock and labor force by province in China from 2002 to 2019, in order to measure the level of inter-provincial factor misallocation in China and its impact on income disparities between regions.

The added value of three major industries and the number of labor force. The annual added value data of the three major industries in China’s provinces are derived from the China Statistical Yearbook over the years. In order to make the annual added value of industries comparable between the years, this paper draws on the methods of Bai and Yang [38] and uses the value-added price index to deflate. The labor force data of the three major industries in each province are mainly from the statistical yearbooks of each province and the “China Economic Database” of the CEIC.

The capital stock of three major industries. In 2002, the data of the physical capital stock of the three major industries in China’s provinces were mainly derived from the data of the physical capital stock of the three major industries in China calculated by Xu et al. [39], and on this basis, the perpetual inventory method was used to calculate the physical capital stock of the three major industries in China’s provinces from 2003 to 2019. According to the Dividing Basis of Three Industries in 2012, the fixed asset investment data of the three major industries in each province are obtained by adding up the fixed asset investment data of relevant industries, and the depreciation rate of physical capital is set at 9.6% with reference to the method of Zhang et al. [40]. The data on fixed asset investment of the three major industries in each province are mainly obtained from the “China Fixed Asset Investment Database” in the EPS database, which is obtained after deflating its annual data using the fixed asset investment price index.

### The assessment results and analysis of factor misallocation among China’s three major industries

#### Assessment results of factor misallocation between China’s three major industries

Tables 1–3 are the total factor misallocation, capital misallocation and labor misallocation between the three major industries in various provinces in China from 2002 to 2019, respectively.

**Table 1:** Total misallocation between the three major industries in each province from 2002 to 2019 (%).

Province	2003	2005	2007	2009	2011	2013	2015	2017	2019
Beijing	19.46	20.02	15.38	14.59	14.33	11.70	9.72	8.66	9.70
Tianjin	13.40	13.13	13.10	11.81	8.40	5.69	3.45	2.68	2.65
Hebei	26.60	25.72	19.11	17.35	16.77	17.97	18.73	16.48	13.15
Shanxi	32.30	32.55	32.43	32.79	22.39	20.03	13.12	10.46	12.92
Inner Mongolia	41.82	35.03	28.88	27.22	23.82	22.91	22.16	21.67	24.90
Liaoning	36.35	28.58	21.19	19.25	16.57	16.97	16.09	15.56	16.18
Jilin	47.06	28.19	21.54	22.88	23.83	24.71	22.08	19.89	14.94
Heilongjiang	45.92	47.07	31.50	24.32	17.91	17.33	13.00	10.26	10.09
Shanghai	20.09	13.95	12.50	9.73	8.17	7.09	8.27	9.39	13.59
Jiangsu	45.74	40.23	37.48	34.99	32.78	32.98	37.10	39.58	36.44
Zhejiang	17.45	17.68	18.01	18.23	19.89	23.04	24.59	23.89	23.58
Anhui	25.07	25.98	27.89	31.24	33.74	41.38	43.23	36.92	33.65
Fujian	21.57	21.49	20.69	22.69	23.01	26.63	29.39	26.56	19.53
Jiangxi	15.58	17.29	20.43	24.11	23.89	25.79	29.57	33.36	33.57
Shandong	29.35	24.46	20.14	18.39	18.69	18.42	18.85	19.14	17.83
Henan	32.97	31.61	28.50	26.26	20.89	19.94	20.46	19.50	15.65
Hubei	35.81	34.10	31.39	30.67	29.22	30.37	29.60	27.34	24.59
Hunan	29.30	28.90	28.38	30.09	28.42	28.44	27.99	25.93	20.49
Guangdong	42.30	37.87	29.26	27.18	23.80	20.09	17.96	17.03	15.93
Guangxi	36.25	41.14	39.84	32.10	30.99	33.73	31.88	29.28	27.32
Hainan	42.12	33.62	29.34	28.29	31.57	43.66	52.60	56.91	62.94
Chongqing	43.81	37.92	32.98	31.53	28.00	20.49	16.60	17.10	17.97
Sichuan	25.62	29.55	30.35	31.08	27.53	30.32	32.99	32.76	31.08
Guizhou	32.21	35.25	36.57	37.75	37.03	49.14	52.70	52.69	42.79
Yunnan	61.95	64.04	54.08	49.11	40.66	37.68	37.02	32.49	28.16
Shaanxi	31.18	28.28	31.69	33.42	24.72	21.20	27.14	20.89	19.07
Gansu	44.94	39.08	44.66	41.44	36.22	36.06	32.23	26.65	24.68
Qinghai	44.78	35.54	25.28	19.63	16.78	13.41	14.99	14.36	16.12
Ningxia	37.70	29.78	23.51	20.09	16.72	28.86	26.65	23.39	19.49
Xinjiang	29.48	26.75	22.90	26.30	23.42	26.94	25.77	27.12	26.88

**Table 2:** Capital misallocation between the three major industries in each province from 2002 to 2019 (%).

Province	2003	2005	2007	2009	2011	2013	2015	2017	2019
Beijing	7.20	7.75	1.00	0.64	2.11	2.49	2.37	2.60	1.90
Tianjin	2.87	3.85	3.60	4.29	6.64	7.41	7.10	7.37	7.09
Hebei	2.70	1.90	1.92	1.38	1.38	2.18	3.00	0.54	2.58
Shanxi	6.53	5.48	6.39	7.55	5.91	6.27	6.95	7.61	8.08
Inner Mongolia	15.39	2.04	3.52	6.25	9.97	10.01	9.22	10.21	8.04
Liaoning	15.55	5.71	3.82	3.14	1.25	1.29	1.78	0.32	0.16
Jilin	25.21	5.52	1.72	1.11	1.09	0.19	0.53	1.10	5.50
Heilongjiang	1.00	0.94	0.53	0.44	1.11	0.20	0.82	1.07	2.80
Shanghai	7.82	6.38	5.53	5.00	5.70	4.94	5.68	6.52	6.04
Jiangsu	16.14	12.20	12.43	10.64	8.25	7.00	7.62	7.04	3.68
Zhejiang	1.15	0.22	1.60	1.61	2.14	3.83	3.17	1.06	0.36
Anhui	1.24	0.51	5.23	8.19	8.75	9.23	8.70	3.99	0.58
Fujian	1.37	0.43	4.10	5.95	5.21	6.82	6.91	3.50	1.35
Jiangxi	6.41	5.73	7.72	9.05	5.22	2.81	3.01	2.82	1.44
Shandong	3.04	3.98	1.86	1.64	1.25	1.41	1.14	1.76	3.19
Henan	1.25	1.22	3.20	3.01	1.52	0.96	1.26	0.95	3.74
Hubei	6.22	5.73	8.77	6.38	3.78	4.24	3.40	1.87	0.21
Hunan	4.33	7.26	10.64	10.97	7.76	6.09	3.63	0.88	3.59
Guangdong	9.05	9.00	3.67	2.89	1.63	1.32	0.07	1.17	2.65
Guangxi	6.61	9.48	10.43	10.85	7.90	8.21	5.23	2.34	0.75
Hainan	32.91	26.03	20.46	16.88	19.78	26.83	32.01	33.98	35.06
Chongqing	8.84	6.99	8.13	3.88	0.22	1.20	2.86	3.99	4.56
Sichuan	2.70	7.00	9.76	11.56	6.82	7.51	7.48	5.07	2.18
Guizhou	4.95	2.50	0.47	2.92	1.99	0.47	6.17	12.58	9.13
Yunnan	3.03	0.32	1.27	3.33	2.95	4.20	6.74	4.45	3.65
Shaanxi	4.89	4.98	1.73	1.10	0.12	1.80	0.50	3.59	4.27
Gansu	12.70	7.06	2.08	0.29	0.17	0.01	1.18	0.34	2.62
Qinghai	6.18	7.94	6.56	6.70	7.10	6.98	5.56	6.42	5.05
Ningxia	2.48	0.06	3.05	3.85	4.34	5.78	5.16	5.56	6.86
Xinjiang	3.01	0.87	0.66	1.02	0.20	1.25	3.65	5.40	2.22

**Table 3:** Labor misallocation between the three major industries in each province from 2002 to 2019 (%).

Province	2003	2005	2007	2009	2011	2013	2015	2017	2019
Beijing	6.22	4.73	7.69	9.39	9.30	8.61	5.38	6.50	10.81
Tianjin	6.40	7.62	8.99	11.02	11.66	10.51	7.00	6.22	10.07
Hebei	18.45	19.59	15.35	15.50	14.82	14.33	10.96	11.07	15.01
Shanxi	25.73	28.41	40.12	49.50	33.12	34.13	25.66	22.55	30.09
Inner Mongolia	16.88	21.48	27.07	31.53	36.05	34.34	26.44	26.11	22.93
Liaoning	16.54	17.51	14.83	15.99	16.45	17.95	14.52	16.01	18.99
Jilin	21.38	19.45	16.97	20.81	22.74	26.27	21.34	17.53	26.19
Heilongjiang	34.71	39.67	28.46	24.17	21.73	19.72	11.30	8.40	5.95
Shanghai	6.37	5.96	10.14	8.40	6.88	2.27	4.67	7.49	9.57
Jiangsu	24.69	24.42	21.05	17.92	15.46	13.29	12.02	11.38	11.75
Zhejiang	10.22	12.03	12.14	12.00	10.99	7.17	6.10	6.34	5.77
Anhui	17.80	20.61	18.40	16.94	16.44	18.85	15.78	13.49	14.61
Fujian	13.71	14.45	11.93	11.66	11.12	9.27	7.68	6.86	7.74
Jiangxi	8.17	7.56	7.94	8.96	10.58	12.76	11.23	11.26	12.67
Shandong	20.81	22.09	19.12	18.29	18.21	17.34	14.81	14.52	16.87
Henan	25.20	28.73	23.12	23.61	19.45	18.21	16.01	17.34	19.68
Hubei	24.62	24.23	20.41	22.51	24.52	25.53	23.55	20.61	19.54
Hunan	20.93	19.32	15.42	15.63	17.48	18.29	19.47	20.89	26.73
Guangdong	23.80	23.93	24.45	25.13	22.45	16.50	13.96	13.40	14.42
Guangxi	25.98	27.22	26.10	20.30	22.43	24.62	26.34	25.88	23.42
Hainan	12.49	11.22	12.23	15.21	13.92	13.65	12.08	10.94	11.57
Chongqing	24.91	22.96	19.38	24.77	27.86	23.66	18.78	16.53	16.01
Sichuan	16.00	16.83	17.54	15.16	16.64	17.39	17.49	16.31	15.06
Guizhou	26.93	33.30	34.77	34.38	36.69	63.17	56.01	38.84	31.56
Yunnan	48.75	53.58	49.69	48.49	42.44	41.70	36.82	36.27	33.08
Shaanxi	25.30	26.50	29.64	31.57	27.09	21.75	38.92	35.45	35.73
Gansu	30.22	30.50	44.45	48.25	44.78	48.44	42.74	36.79	41.11
Qinghai	35.13	34.10	28.43	25.39	24.81	22.46	20.25	20.86	18.50
Ningxia	26.96	23.02	25.62	25.24	22.51	45.21	42.65	38.50	37.36
Xinjiang	26.68	23.49	22.28	27.20	25.34	26.68	22.31	20.95	24.31

(1) Total misallocation. Table 1 shows the total misallocation of factors between the three major industries in 30 provincial-level administrative regions (excluding Hong Kong, Macao, Taiwan and Tibet). Table 1 shows that the misallocation of factors between the three major industries in each province is very heterogeneous. In the eastern region, the degree of factor misallocation between the three major industries in Jiangsu and Hainan was relatively high, and the total misallocation level remained above 30% during the inspection period. The misallocation between the three major industries in Beijing, Tianjin and Shanghai is low, and as of 2019, the misallocation between the three major industries in the above three provinces and cities has been lower than or close to 10%. The level of factor misallocation between the three major industries in Hebei, Fujian, Zhejiang, Guangdong and Shandong remained between 10% and 30% in most years. In the central region, the factor misallocation between the three major industries in Shanxi and Henan was at a high level of more than 20% before 2011, and dropped significantly to less than 20% after 2011. The misallocation of factors between the three major industries of Anhui, Jiangxi, Hubei and Hunan is relatively serious, and most of the years are in a state of serious misallocation of more than 30%. In the western region (Tibet has serious data deficiency and is not within the scope of investigation), the overall factor misallocation between the three major industries in each province is significantly higher than that in the eastern region and the central region, and the misallocation level of factors between the three major industries in Chongqing, Shaanxi, Qinghai and Ningxia is relatively low in the western region, and as of 2019, the factor misallocation level of the above four provinces and cities has been less than 20%. In most of the years, the factor misallocation in Xinjiang and Inner Mongolia hovered between 20% and 30%, and the level of factor misallocation in Guangxi, Sichuan, Guizhou, Yunnan and Gansu was relatively high, basically above 30%. Among them, Guizhou and Yunnan have the most serious misallocation, and the misallocation level has even exceeded 50% in some years. The factor misallocation between the three major industries in the three provinces in the northeast of China was serious at first, and then showed a trend of improvement year by year, and as of 2019, the factor misallocation in the three northeastern provinces has all dropped to between 10% and 20%.

From the perspective of the change trend of factor misallocation between the three major industries in each province, the misallocation of factors between the three major industries in the Bohai Rim region (Beijing, Tianjin, Hebei, Shandong), the departments and provinces in the northwest region (Shaanxi, Inner Mongolia, Gansu, Qinghai and Shanxi), the northeast region (Liaoning, Jilin, Heilongjiang), Henan, Hubei and Chongqing in the central region, Guangdong in the south China, Yunnan and Guangxi in the southwest region have shown a significant decline trend year by year. The efficiency of factor allocation between their three major industries has improved significantly year by year. The misallocation between the three major industries of Shanghai, Jiangsu and Ningxia took 2013 as the inflection point, showing a U-shaped trend of first falling and then rising. The misallocation of factors between the three major industries of Zhejiang, Anhui, Fujian, Jiangxi, Sichuan and Guizhou is increasing year by year.

**Fig 1:**
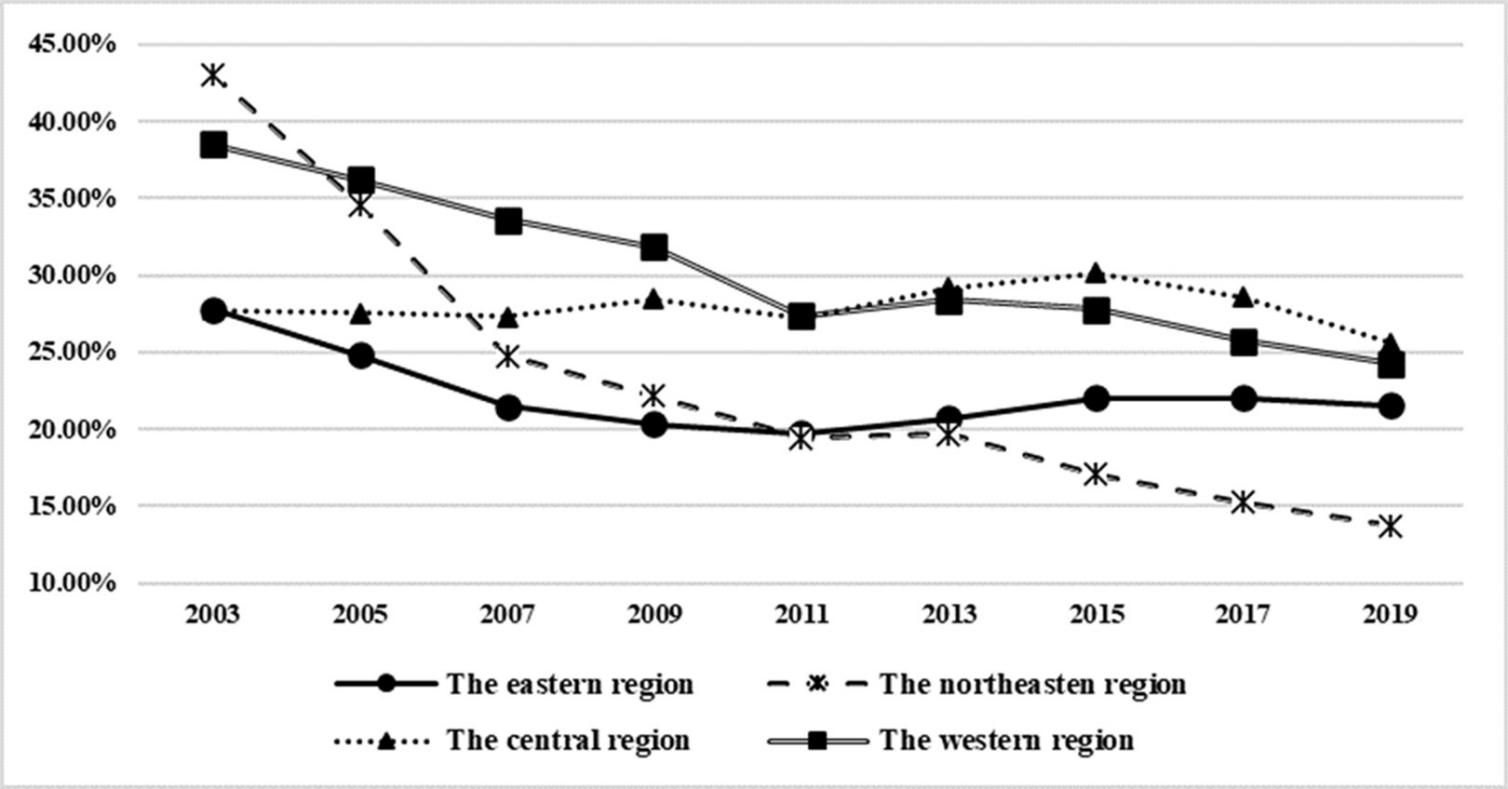
Annual change of the average of total misallocation between the three major industries in each region from 2002 to 2019. Data source: Calculated by the authors based on the data in Tables 1–3.

(2) Capital misallocation. It can be seen from Table 2 that among the capital factor misallocation between the three major industries in various provinces in China, the capital misallocation in the eastern region and Hainan is seriously high, and it shows a U-shaped evolution trend, and its capital misallocation is still as high as 35.06% as of 2019; The capital misallocation in Beijing, Jiangsu, Shandong and Guangdong showed a downward trend year by year, of which the capital misallocation in Beijing, Shandong and Guangdong has been below 10% since 2002, and then has dropped all the way to below 4%, and the degree of capital misallocation in Jiangsu was serious at first, and then declined rapidly to 3.68% in 2019. In the central region, the capital misallocation of all provinces is basically at a low level below 10%, among which, the capital misallocation in Anhui, Henan and Hunan shows an inverted U-shaped trend. Shanxi’s capital misallocation basically remained between 5% and 8%, and increased steadily from 2002 to 2019; The level of capital misallocation in Jiangxi and Hubei is low and gradually declining. In the western region, the capital misallocation in Inner Mongolia was relatively serious at first, and then gradually decreased to around 2010, and then gradually increased, showing a significant U-shaped change trend. The capital misallocation of Chongqing, Guizhou, Shaanxi and Xinjiang also showed a U-shaped trend, but their capital misallocation remained at a low level. The capital misallocation level of Guangxi, Ningxia and Sichuan is low, and it shows an inverted U-shaped trend. Capital misallocation of Gansu initially exceeded 10%, and then declined year by year. Capital misallocation in the three northeastern provinces except for Jilin in the first year, the capital misallocation of Heilongjiang and Liaoning are at a low level, and the capital misallocation in the three provinces has very different trends, Liaoning shows a downward trend year by year, Jilin shows a U-shaped change trend of first falling and then rising, and Heilongjiang shows a steady evolution trend without significant increase or decrease.

(3) Labor misallocation. In the eastern region, the labor misallocation degree of Hebei, Jiangsu, showed an inverted U-shaped trend, and the other four provinces showed a downward trend as a whole. The labor misallocation levels of Beijing, Tianjin, Shanghai, Zhejiang and Fujian are relatively low, most of the years are below 10%, Fujian shows a significant downward trend year by year, the remaining three provinces and cities basically show an inverted U-shaped trend of rising first and then falling. In the central region, the level of labor misallocation in Shanxi, Henan, Hubei and Hunan is relatively high, and most of the years are in a state of misallocation of more than 20%, among which Shanxi shows an inverted U-shaped trend of rising first and then falling, Henan and Hubei show a downward trend year by year, and Hunan shows a U-shaped change trend. The level of labor misallocation in Anhui and Jiangxi is relatively low, but the trend is different, Anhui’s labor misallocation shows a downward trend year by year, and although Jiangxi’s labor misallocation is basically at a level of about 10%, it is increasing year by year. In the western region, the labor misallocation level of all provinces is significantly higher than that in the eastern and central regions, with Guizhou, Yunnan, Shaanxi, Gansu, Qinghai, Ningxia and Xinjiang having a labor misallocation of more than 30% in most of the time, while Guangxi, Chongqing and Inner Mongolia remaining between 20% and 30% in most of the time, and the labor misallocation in Sichuan is relatively low in the western region, but it is always above 10%. In the northeast, Heilongjiang initially had the highest level of misallocation, but the downward trend was the strongest, falling from 34.71% to 5.95% as of 2019; The labor misallocation in Liaoning is relatively low, basically remaining between 10%-20%, and showing a U-shaped development trend. Labor misallocation in Jilin basically remained at a high level between 20% and 30%, showing a steady evolution.

(4) The change trend and structural characteristics of factor misallocation. It can be seen from Fig 1 that the trend of factor misallocation between the three major industries in the eastern, central, western and northeastern provinces is roughly decreasing year by year, but its decline rate is different from the local change trend. The Northeast region initially had the highest level of factor misallocation, but its decline range and rate were the fastest, and as of 2019, the average factor misallocation between the three major industries in its provinces was already at the lowest level in all regions. The western region was second only to the northeast at first, and then declined, but its decline rate was significantly lower than that of the northeast region, and the misallocation level was only lower than that of the western region in 2019. The factor misallocation between the eastern region and the central region was similar in the initial years, and then showed a discrete trend, with the eastern region decreasing year by year from 2002 to 2009, and the central region fluctuating and increasing from 2002 to 2015, which led to a widening gap in factor misallocation between central and eastern regions. After 2009, the level of factor misallocation in the eastern region began to rise, and there was a convergence trend with the level of factor misallocation in the central region after 2015, as of 2019, the factor misallocation gap between the central and eastern regions has been less than 5%. It can be seen that there are great differences in resource endowment, industrial structure and market development level in different regions and provinces in China, resulting in significant heterogeneity in the misallocation level and evolution trend of factors among the three major industries in each province. The discrete evolution and asynchronous improvement of factor misallocation levels within different provinces are likely to have a negative impact on income disparities between regions, between industries and within industries.

**Fig 2:**
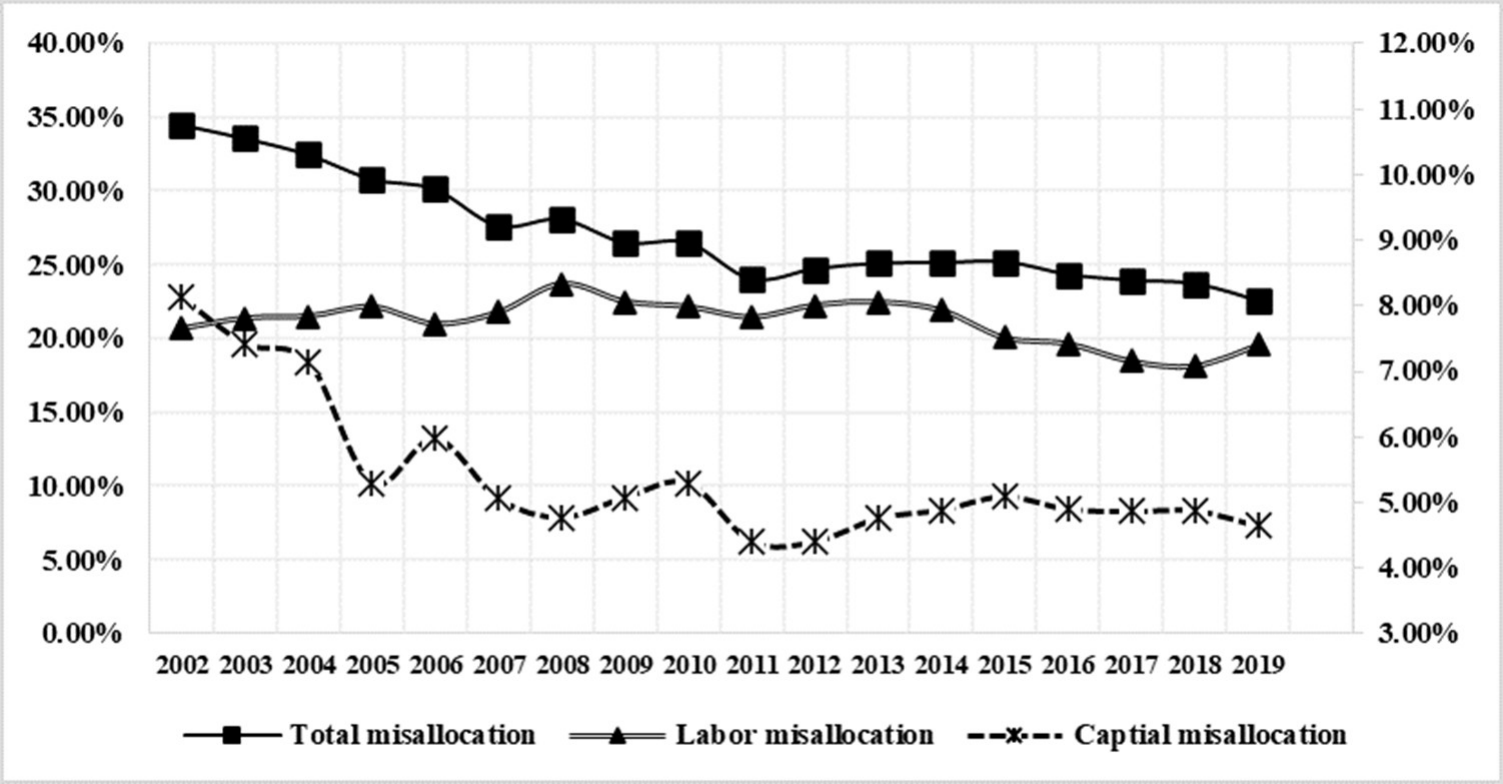
Annual changes in the average level of total misallocation, capital misallocation and labor misallocation between the three major industries in each province from 2002 to 2019. Data source: Calculated by the authors based on the data in Tables 1–3.

From the perspective of subdivision factor misallocation (as can be seen from Fig 2), the average of labor misallocation is significantly higher than the average of capital misallocation, the factor misallocation between the three major industries in each province is mainly caused by labor misallocation, and the contribution of the factor misallocation between the three major industries in each province is increasing year by year. Therefore, in the following analysis of the impact of factor misallocation changes among China’s three major industries on the pattern of income distribution, we should pay particular attention to the impact of labor misallocation changes. The capital misallocation showed an annual downward trend from 2002 to 2005 (see right axis for specific values), and then fluctuated steadily, basically remaining between 4% and 5%.

#### The input status of the three major industrial factors in various provinces in China

This paper uses the above theoretical model to calculate the ratio of labor and capital input li, ki of the three major industries in each province in a distorted state (that is, the actual state), and the proportion of factor input of each industry in a non-distorted state l^*^_i_, k^*^_i_, by calculating the ratio of factor input in the distorted state and the undistorted state, *pli* = *l*_*i*_/l^*^_i_ and *pki* = *k*_*i*_ / k*_i_, so as to obtain the degree of excessive or insufficient input of the three major industrial factors in each province. The closer *pli* and *pki* to 1, the more ideal the actual state of labor and capital input in the industry, greater than 1 means that the factor input of the industry is excessive, and less than 1 means that the factor input of the industry is insufficient.

As can be seen from Table 4, taking the recent situation of 2019 during the period under review as an example, the input of labor factors in the primary industry (agriculture) in all provinces in China has been significantly excessive, and a large number of redundant labor has settled in the agricultural field, which is urgent to accelerate the transfer to the secondary and tertiary industries through the flow and allocation mechanism of innovative factors, in order to further optimize the social division of labor. Except for Beijing, Tianjin, Shanxi and Shaanxi, where the primary industry capital investment is in a state of significant excess, and the primary industry capital investment in Jilin and Ningxia is basically in an effective and reasonable state (factor input deviates from the effective level by less than 10%), the primary industry capital investment in the other provinces is in a state of significant inadequacy. Therefore, in the future, it is necessary to increase capital investment in agriculture, and promote the rapid transformation of agriculture in various provinces to mechanization, automation and digitalization by improving the capital intensity of agriculture, in order to help alleviate the inefficient input of agricultural capital factors. In 2019, the input of labor factors in the secondary and tertiary industries in all provinces in China was in a state of inadequacy, which further confirmed the fact that China urgently needs to optimize the social division of labor and labor employment mechanism to revitalize the flow and allocation of redundant agricultural labor to the secondary and tertiary industries. From the perspective of the capital factor investment status of the secondary and tertiary industries, the capital investment of the secondary industry in Hebei, Inner Mongolia, Jilin, Zhejiang, Anhui, Henan, Hubei, Hunan and Xinjiang was very close to the effective state in 2019, and the capital factor investment in the secondary industry in Liaoning, Heilongjiang, Jiangsu, Jiangxi, Shandong and Gansu was in a serious state of excess, and the capital input of secondary industry in remaining 14 provinces is in a state of serious inadequacy. It can be seen that the input efficiency of secondary industry capital factors in various provinces in China is heterogeneous. In 2019, the problem of excessive capital input in the tertiary industry in various provinces in China was more serious than that in the secondary industry. The investment status of tertiary industry capital factors in Beijing, Tianjin, Hebei, Inner Mongolia, Jilin, Jiangsu and Gansu is basically close to the effective level, the tertiary industry capital factors in Shanxi and Heilongjiang are in a state of significant underinvestment, and the tertiary industry capital factors in the remaining 21 provinces are in a significant state of excess. It can be seen that the misallocation of factors between China’s three major industries is mainly caused by the contradiction between excessive agricultural labor input and insufficient labor input in the secondary and tertiary industries, and the contradiction between insufficient input of agricultural capital factors and excessive capital input in the secondary and tertiary industries (especially the tertiary industry).

**Table 4:** The input status of three major industrial factors in China’s provinces in 2019: excessive or insufficient.

Industry	Primary industry		Secondary industry		Tertiary industry	
Province	pli	pki	pli	pki	pli	pki
Beijing	12.2135	6.7483	0.3791	0.4095	0.5759	1.0007
Tianjin	8.7040	4.2656	0.3820	0.6507	0.5648	0.9054
Hebei	2.9413	0.7176	0.4452	1.0602	0.4973	1.0980
Shanxi	7.1597	3.2207	0.2327	0.7040	0.3351	0.7833
Inner Mongolia	3.3031	0.8881	0.2004	1.0875	0.4947	0.9712
Liaoning	3.1732	0.4453	0.4036	1.1023	0.5870	1.1597
Jilin	3.5793	1.0621	0.2119	1.0028	0.5566	0.9625
Heilongjiang	2.0053	0.5191	0.6509	1.4865	0.7243	0.8059
Shanghai	7.8977	0.9885	0.5608	0.7399	0.5573	1.2454
Jiangsu	2.3918	0.2129	0.6691	1.3709	0.5189	1.0876
Zhejiang	2.4531	0.4138	0.7583	0.9073	0.5594	1.3919
Anhui	2.3268	0.4122	0.3471	0.9756	0.6423	1.5459
Fujian	2.5234	0.5697	0.4498	0.7202	0.6946	1.6070
Jiangxi	2.2708	0.4005	0.4338	1.2273	0.6203	1.2163
Shandong	3.2283	0.6071	0.4603	1.1762	0.4254	1.0161
Henan	3.1041	0.7609	0.3608	1.0028	0.4279	1.1488
Hubei	2.7253	0.4401	0.3099	0.9893	0.6177	1.4140
Hunan	3.2516	0.6867	0.2608	0.9218	0.4129	1.2749
Guangdong	3.6059	0.5292	0.4840	0.8185	0.5036	1.3811
Guangxi	2.5782	0.4433	0.2561	0.9204	0.5383	1.5496
Hainan	1.3934	0.0926	0.4213	0.6360	0.9878	2.6192
Chongqing	3.2192	0.7127	0.2998	0.6925	0.5733	1.4978
Sichuan	2.3926	0.4063	0.4279	0.8831	0.5025	1.5824
Guizhou	2.8886	0.3192	0.2421	0.5426	0.4312	2.1753
Yunnan	3.1916	0.5511	0.1904	0.5582	0.6009	2.0078
Shaanxi	4.6726	1.1926	0.1518	0.4590	0.5570	1.7717
Gansu	4.0125	0.7504	0.2457	1.1253	0.2963	0.9927
Qinghai	3.2499	0.6782	0.2830	0.8834	0.6250	1.3113
Ningxia	4.6309	1.0706	0.1702	0.8244	0.4864	1.1760
Xinjiang	2.5194	0.4464	0.2325	1.0077	0.8338	1.4596

Compared with the research conclusions of Bai [35], the difference between the research conclusions of this paper and that of Bai [35] is mainly in the labor allocation state of the Tertiary sector of the economy. According to the research of Bai [35], the labor force allocated in China’s Primary sector of the economy is more than that in the undistorted state, and the labor force in the Tertiary sector of the economy is mostly more than that in the undistorted state, while the labor force allocated in the Secondary sector of the economy is significantly lower than that in the undistorted state, However, the proportion of configuration distortion is generally decreasing. However, the sample interval studied by Bai [35] is from 1978 to 2011, and the sample data is very outdated and cannot reflect the latest industrial development trends and labor factor allocation in China. The research conclusion of this article can better reflect the latest industrial development and factor allocation efficiency status in China.

(5) Analysis of the change trend and convergence of factor misallocation between the three major industries in each province. Table 5 shows the variance and coefficient of variation of the total misallocation, capital misallocation and labor misallocation between the three major industries in each province. It can be seen that the variance and coefficient of variation of the total misallocation in each province have a U-shaped change structure with 2011 as the inflection point, that is, before 2011, there is a convergence trend between the three major industries in China’s provinces, but after 2011, it shows a reverse convergence trend. The variance and coefficient of variation of labor misallocation diverged before 2009, and then diverged significantly from 2011 to 2013 after a brief convergence from 2009 to 2011. After 2013, it showed significant convergence characteristics. The variance and coefficient of variation of capital misallocation are more consistent with the change trend of total misallocation, showing a U-shaped change structure, but the inflection point value is 2 years earlier than that of total misallocation. Similar to total misallocation, capital misallocation converges before the inflection point and diverges significantly after the inflection point.

**Table 5:** The variance and coefficient of variation of factor misallocation between the three major industries over the years.

Year	2003	2005	2007	2009	2011	2013	2015	2017	2019
Total misallocation									
Variance	0.0126	0.0107	0.0085	0.0076	0.0064	0.0105	0.0140	0.0148	0.0135
Coefficient of variation	0.0376	0.0347	0.0307	0.0285	0.0265	0.0419	0.0557	0.0618	0.0601
Labor misallocation									
Variance	0.0090	0.0108	0.0110	0.0126	0.0093	0.0179	0.0157	0.0104	0.0093
Coefficient of variation	0.0420	0.0486	0.0504	0.0561	0.0435	0.0795	0.0783	0.0559	0.0474
Capital misallocation									
Variance	0.0054	0.0026	0.0020	0.0017	0.0017	0.0026	0.0033	0.0040	0.0039
Coefficient of variation	0.0731	0.0488	0.0395	0.0334	0.0395	0.0553	0.0647	0.0813	0.0843

### The impact of the factor misallocation on the income distribution pattern between the three major industries in China

Table 6 shows the impact of the inter-industrial factor misallocation between the three major industries in each province of China on the income gap within the three major industries. Among them, in terms of the impact of total misallocation, the S-values of the income gap within the primary, secondary and tertiary industries in 2003 were 7.4918, 1.1734 and 0.9083, respectively, indicating that in the first year, correcting the factor misallocation between the three major industries in China’s provinces could only narrow the income gap within the tertiary industry and explain 9.17% of the income gap within the tertiary industry; Correcting the misallocation of factors between the three major industries in various provinces in China has a widening effect on the income gap within the primary industry and the secondary industry, which can widen the income gap within the primary and secondary industries by 649.18% and 17.34% respectively. This is caused by the fact that the factor input ratio of primary and secondary industries in high-income provinces deviates from the effective state more seriously than that in low-income provinces. After correcting the total misallocation of factors, the per capita income of the primary and secondary industries in high-income provinces increased by a higher rate than that in low-income provinces, thereby widening the income gap between the primary and secondary industries. After 2003, the S-value of the income gap between the primary, secondary and tertiary industries basically showed a U-shaped trend of first falling and then rising around 2011, which was highly consistent with the U-shaped trend of factor misallocation dispersion. As of 2019, the S values of the income gap within the three major industries after correcting the total misallocation of factors between the three major industries in each province were 3.3672, 1.3864 and 0.9147, respectively. During the inspection period, the S value of the income gap between the primary industry and the secondary industry is always greater than 1, and the S value of the income gap within the tertiary industry is always less than 1, and the total misallocation of factors between the three major industries in 2019 can explain 8.53% of the income gap within the tertiary industry.

**Table 6:** The impact of the factor misallocation between China’s three major industries on the income distribution gap within the three major industries.

Year	2003	2005	2007	2009	2011	2013	2015	2017	2019
Correct total misallocation									
Inside the primary industry	7.4918	4.8916	3.8334	3.6816	3.5479	2.8795	3.2287	3.2679	3.3672
Inside the secondary industry	1.1734	1.1271	1.2403	1.1213	1.2023	1.1633	1.3568	1.4453	1.3864
Inside the tertiary industry	0.9083	0.9140	0.8384	0.8333	0.8669	0.9238	0.9454	0.9394	0.9147
Only correct capital misallocation									
Inside the primary industry	5.9394	3.4713	2.2794	2.0381	1.9722	1.8341	2.5160	2.4086	2.0704
Inside the secondary industry	1.5044	1.5661	1.5319	1.4001	1.3514	1.3150	1.6166	1.7402	1.9116
Inside the tertiary industry	1.1188	0.9926	0.9372	0.9665	1.0445	1.1063	1.1552	1.1915	1.1854
Only correct labor misallocation									
Inside the primary industry	2.0889	2.1081	2.8960	3.2215	3.1123	2.6250	2.8241	3.3261	3.4982
Inside the secondary industry	0.9037	0.7852	0.8176	0.7791	0.8842	0.8470	0.8687	0.8997	0.8202
Inside the tertiary industry	0.8513	0.9376	0.9026	0.8697	0.8380	0.8522	0.8261	0.8058	0.7936

In terms of the impact of capital misallocation, the S value of the income gap within the primary, secondary and tertiary industries was initially greater than 1, indicating that only correcting the capital misallocation between the three major industries in each province had an effect on the expansion of the income gap within the three major industries in the initial year, and the S value of the income gap within the primary industry and the income gap within the secondary industry were greater than 1 during the survey period. The S-value of the income gap within the tertiary industry decreased year by year from 2002 to 2011, and was less than 1 from 2005 to 2009. It showed an upward trend from 2011 to 2015, and then decreased year by year. The S-value of the income gap within the primary and secondary industries is similar to that of the income gap within the tertiary industry, but the inflection point year is slightly different. It can be seen that the trend of the S-value change of the income gap within the three major industries is basically consistent with the trend of the dispersion level of capital misallocation between the three major industries in each province. Therefore, in the process of correcting the structural misallocation of internal factors by each province independently, it is likely to lead to the expansion of the level of factor misallocation between regions and the degree of dispersion of total factor productivity, which is obviously not conducive to the coordinated development of inter-regional development and the narrowing of income gap. The above empirical results also verify this conclusion.

Labor misallocation. In the first year, the S value of the income gap within the primary industry was 2.0889, that is, correcting the labor misallocation between the three major industries increased the income gap within the primary industry by 108.89%. The S values of the income gap within the secondary industry and the tertiary industry were 0.9037 and 0.8513 respectively in the first year, that is, the labor misallocation between the three major industries could explain 9.63% and 14.87% of the income gap within the secondary industry and within the tertiary industry, respectively. From 2002 to 2019, the S value of the income gap within the secondary and tertiary industries when only correcting labor misallocation was always less than 1. As of 2019, the S values of the income gap within the secondary and tertiary industries were 0.8202 and 0.7936, respectively, that is, correcting the labor misallocation in 2019 can reduce the income gap within the secondary and tertiary industries 17.98% and 20.64% respectively, that is, correcting labor misallocation can significantly narrow the income gap within the secondary and tertiary industries.

Yang and Bai [28] used a similar method to this article to measure the impact of factor misallocation between China’s agricultural and non agricultural sectors on the income gap within the agricultural and non agricultural sectors, which found that correcting the factor misallocation between China’s agricultural and non agricultural sectors can significantly reduce the income gap within the agricultural sector, but expand the income gap within the non agricultural sector. This paper further refines the non-agricultural sector into the Secondary sector of the economy and the Tertiary sector of the economy. Through investigating the impact of correcting the factor misallocation among China’s three major industrial sectors on the income gap within the three industries, it is found that after correcting the factor misallocation among the three industries, the income gap within the agricultural and Secondary sector of the economy sectors has been widened, but the income gap within the Tertiary sector of the economy has been narrowed. That is, the research conclusions of this paper are further refined on the basis of the research conclusions of Yang and Bai [28]. According to the research of this paper, we can further draw the following conclusions: the narrowing effect of correcting the factor misallocation on the internal income gap of the non-agricultural sector is mainly reflected in the internal Tertiary sector of the economy sector, and the internal income gap of the Secondary sector of the economy sector is still expanding.

Correcting the total misallocation between the three major industries can significantly reduce the income gap between provinces before 2017, but after 2017, its impact on the income distribution gap become expansion. From the perspective of factor misallocation, the impact of correcting the capital misallocation and labor misallocation on the income distribution gap between provinces is also similar to that of total misallocation, gradually turning from narrowing to expanding. The same is true of correcting the factor misallocation between the three major industries on the income gap between the industries, that is, from 2002 to 2013, it can significantly narrow the income gap between industries, but then the S value of the income gap between industries gradually widens. The impact of correcting the misallocation of total factors, capital misallocation and labor misallocation between the three major industries in each province on the income gap between provinces and industries has gradually turned from narrowing to expanding. As of 2019, correcting the total misallocation between the three major industries in each province will increase the income gap between provinces by 17.64% and the income gap between industries by 184.58%. The results of the impact of the factor misallocation between China’s three major industries on the income disparities between provinces and industries are shown in Table 7.

**Table 7:** The impact of the factor misallocation between China’s three major industries on the income disparities between provinces and industries.

Year	2003	2005	2007	2009	2011	2013	2015	2017	2019
Correct total misallocation									
Among provinces	0.6952	0.7354	0.7168	0.7738	0.8325	0.8847	0.9106	0.9883	1.1764
Among industries	0.1762	0.1907	0.1556	0.1737	0.2369	0.6891	1.8637	3.2501	2.8458
Correct capital misallocation									
Among provinces	0.9888	0.9914	0.9713	0.9572	0.9541	0.9729	1.0172	1.0704	1.1537
Among industries	0.7442	0.9460	0.6911	0.5856	0.4673	0.9482	2.4049	4.3597	4.1173
Correct labor misallocation									
Among provinces	0.6997	0.7494	0.8310	0.9109	0.9788	0.9782	1.0637	1.2379	1.4825
Among industries	0.3657	0.3937	0.4985	0.5932	0.6837	0.7734	0.9395	1.3159	1.6438

### Further analysis of inter-provincial factor misallocation and inter-provincial income gap

As can be seen from the above research, as of 2019, correcting the misallocation between three major industries within provinces has been unable to narrow the income gap between provinces and between industries. As far as the income gap within the industry is concerned, the provinces can only narrow the income gap within the tertiary industry by correcting the misallocation of factors between the three major industries in their own ways. In the context of the current accelerated construction of the new development pattern of "dual circulation" and the acceleration of the construction of the "domestic unified market", the question we need to further consider is, if we promote the reflow and reallocation of production factors by breaking through the restrictions of inter-provincial boundaries and reallocating them within a larger spatial range, will the income gap between regions in China be narrowed while improving the efficiency of inter-provincial factor allocation? To answer this question, this paper continues to measure the level of inter-provincial factor misallocation in China from 2002 to 2019 and its impact on inter-provincial income disparity. The empirical results are shown below.

### Estimation results and analysis of inter-provincial factor misallocation level in China

Table 8 shows that the average annual misallocation level of inter-provincial factor misallocation in China was 8.27% from 2002 to 2019, which was much lower than the misallocation of the three major industrial factors. And from the perspective of sub-factor misallocation, the average annual misallocation of capital factors reached 1.18%, and the average annual level of labor misallocation was 6.73%, which shows that China’s inter-provincial factor misallocation is also mainly caused by labor misallocation, correcting the inter-provincial misallocation of labor factors is important to improve the efficiency of China’s inter-provincial factor allocation. In the investigation of the impact of inter-provincial factor misallocation between provinces on the income gap between provinces, special attention should be paid to the impact of labor misallocation.

**Table 8:** Inter-provincial factor misallocation level in China in 2019 (%).

Year	Total misallocation	Labor misallocation	Capital misallocation	Year	Total misallocation	Labor misallocation	Capital misallocation
2002	8.47	7.70	0.87	2011	8.17	6.64	1.10
2003	8.73	7.96	0.80	2012	8.05	6.44	1.17
2004	9.50	8.63	0.80	2013	7.59	5.92	1.18
2005	9.03	8.00	0.87	2014	7.36	5.63	1.20
2006	8.72	7.60	0.89	2015	6.98	5.12	1.27
2007	9.29	8.03	0.96	2016	7.07	5.05	1.36
2008	9.12	7.80	1.02	2017	7.23	5.04	1.45
2009	9.10	7.69	1.03	2018	7.72	5.37	1.93
2010	8.67	7.22	1.06	2019	8.13	5.27	2.33

**Fig 3:**
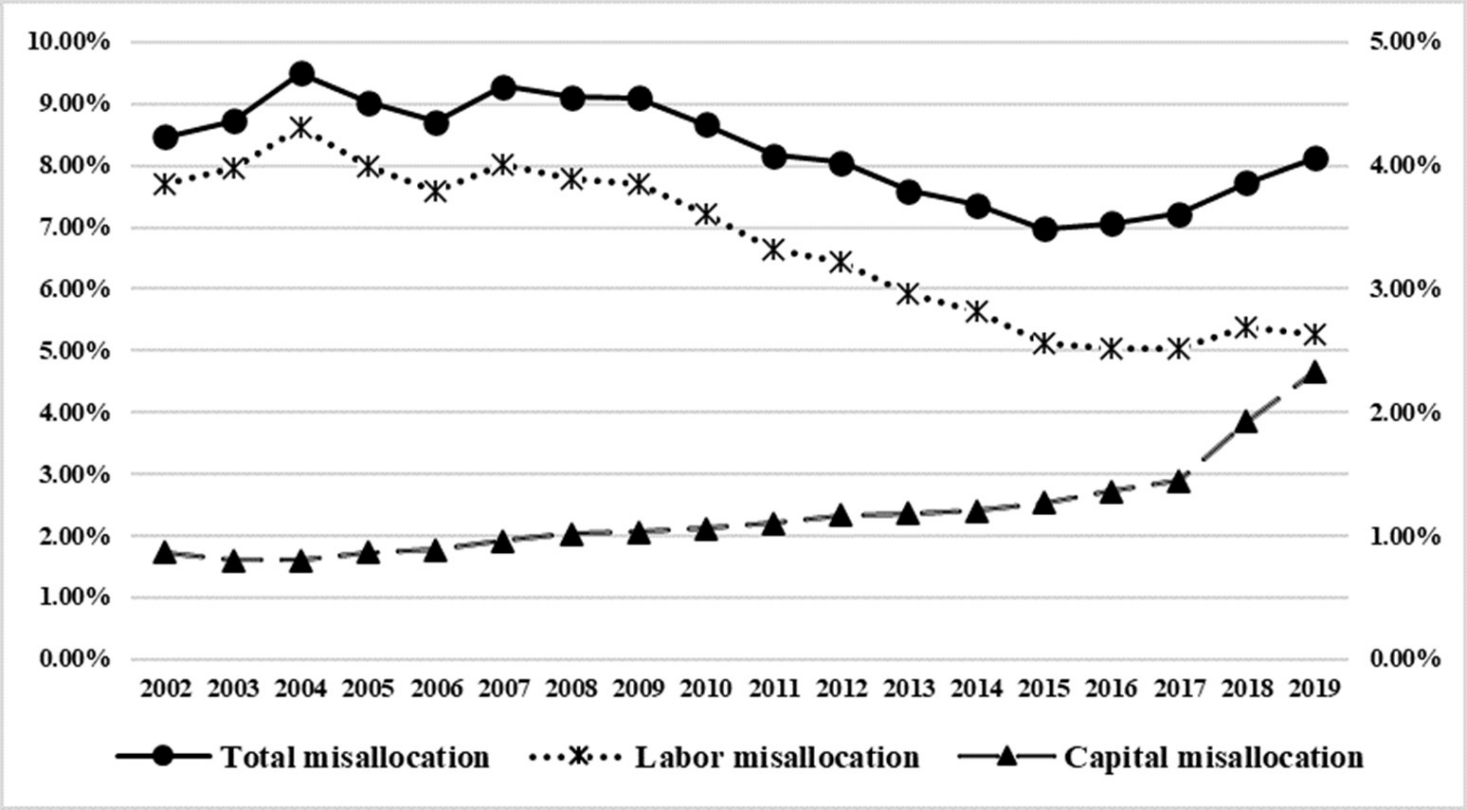
Inter-provincial factor misallocation in China from 2002 to 2019.

From the perspective of the change trend (Fig 3), China’s inter-provincial factor misallocation showed a development trend of first declining and then rising, and its total factor misallocation showed a significant decline trend from 2002 to 2015, and then went all the way up, and the change trend of labor misallocation was similar to the change trend of total misallocation. Although the level of capital misallocation is low (the value is the right axis), it has shown a significant upward trend, and as of 2019, its capital misallocation level has risen to 2.33%. Therefore, the problem of capital misallocation between provinces in China also needs attention.

### The impact of inter-provincial factor misallocation in China on inter-provincial income gap

It can be seen from Table 9 that in terms of the impact of inter-provincial total factor misallocation, the S value of inter-provincial income gap in 2003 was 0.1995, indicating that inter-provincial factor misallocation has strong explanatory power and can explain 80.05% of the inter-provincial income gap. Subsequently, although the S value of inter-provincial income gap increased year by year, as of 2019, correcting the total misallocation of factors between provinces can still explain 51.48% of the income gap between provinces (that is, correcting the total misallocation of factors between provinces can significantly reduce the income gap between provinces by 51.48%). In terms of the impact of inter-provincial capital misallocation, the S value of the inter-provincial income gap after correcting the capital misallocation was 0.9616, and the explanatory power of inter-provincial capital misallocation on inter-provincial income gap was only 3.84%, which was much lower than the explanatory strength of the inter-provincial total misallocation, which is closely related to the low level of inter-provincial capital misallocation in China. Without the impact of superimposed labor misallocation, only correcting the capital misallocation has little effect on China’s inter-provincial income gap, which narrowed China’s inter-provincial income gap from 2002 to 2005, and then the S value changed from less than 1 to greater than 1, that is, only correcting the inter-provincial capital misallocation will not narrow the inter-provincial income gap, but will widen the inter-provincial income gap, up to 2019, the S value of the income gap between provinces has increased to 2.1221, that is, all other things being equal, only correcting the capital misallocation between provinces will increase the income gap between provinces in China by 112.21% in 2019. Only correcting labor misallocation, the S-value was significantly lower than the S-value when correcting the total misallocation and only correcting the capital misallocation from 2002 to 2019, and the S-value during the survey period was always less than 0.25%, that is, the explanatory power of inter-provincial labor misallocation on China’s inter-provincial income gap is always higher than 75%, indicating that labor misallocation has strong explanatory power for China’s inter-provincial income gap. Even in the 2019, correcting labor misallocation alone was able to reduce China’s inter-provincial income gap by 87.68%.

**Table 9:** The impact of inter-provincial factor misallocation on income distribution gap between provinces in China from 2002 to 2019.

Year	2003	2005	2007	2009	2011	2013	2015	2017	2019
Correct total misallocation	0.1995	0.2239	0.2485	0.2345	0.2583	0.2569	0.2997	0.3731	0.4852
Only correct capital misallocation	0.9616	0.9789	1.0446	1.1328	1.1288	1.1768	1.2473	1.3997	2.1221
Only correct labor misallocation	0.1860	0.2037	0.2126	0.1911	0.2180	0.2268	0.2487	0.2467	0.1232

It can be concluded that the regional integration and factor allocation improvement within each province can no longer narrow the income gap between provinces and industries in China, and cannot solve the main contradiction of China’s current imbalance and insufficient development, but should carry out inter-regional integration development on a larger spatial scale (such as inter-province) and promote the flow of factors across provinces and efficient and reasonable allocation, so as to correct the distortion of inter-provincial factor allocation. Only in this way can we effectively narrow the income gap between provinces, help China build a total factor productivity-driven economic growth model, and achieve a fair and just income distribution pattern, which ultimately contributes to the smooth realization of the common prosperity development goal.

## Conclusion and enlightenment

This paper measures the degree of factor misallocation between the three major industries in China’s provinces from 2002 to 2019, and focuses on the impact of the change of factor allocation efficiency between the three major industries on the income distribution pattern. It is found that: (1) There is a serious factor misallocation between the three major industries in each province, and although the factor misallocation shows a downward trend year by year, as of 2019, the average of factor misallocation between the three major industries in China’s provinces is still as high as 22.53%. From the perspective of subdivided factors, the misallocation of factors between the three major industries in China’s provinces is mainly caused by labor misallocation, and from 2002 to 2019, the average annual contribution of labor misallocation to the total misallocation of factors between the three major industries in China’s provinces is as high as 80.24%. Moreover, after the financial crisis in 2008, the factor misallocation between the central and western provinces became more serious, and the factor misallocation in the north-east region was rapidly alleviated. The variance and coefficient of variation of the total misallocation in each province showed a U-shaped change structure with 2011 as the inflection point, that is, before 2011, there was a convergence trend in the total misallocation between the three major industries in China’s provinces, but after 2011, it showed a reverse convergence trend. (2) There is strong heterogeneity in explanatory power of factor misallocation between the three major industries in China’s provinces to income gap in different dimensions. Among them, correcting the misallocation of total factors between the three major industries in China’s provinces can only narrow the income gap within the three major industry, and its explanatory power is not high, and the total misallocation of factors between the three major industries in 2019 can only explain 8.53% of the income gap within the tertiary industry, and correcting the misallocation of total factors between the three major industries in China’s provinces has a widening effect on the income gap within the primary and secondary industry. Correcting the labor misallocation can significantly narrow the income gap within the secondary and tertiary industries, and have a widening effect on the income gap within the primary industry. As of 2019, the S values of the income gap within the primary, secondary and tertiary industries were 3.4982, 0.8202 and 0.7936, that is, in 2019, correcting the labor misallocation can narrow the income gap within the secondary and tertiary industries by 17.98% and 20.64% respectively, and widen the income gap within the primary industry by 249.82%. Only correcting the capital misallocation has a widening effect on the income gap within the primary, secondary and tertiary industries. (3) The impact of correcting the impact of the total misallocation between three major industries on the income gap between industries and provinces is delimited by 2013 and 2017 respectively. Before 2013 and 2017, the income gap between industries and provinces can be significantly reduced, but after 2013, the income gap between industries is expanded. After 2017, its influence on the income distribution gap between provinces has changed from narrowing to expanding. The influence of only correcting the labor misallocation and only correcting the capital misallocation on the inter-industrial and inter-provincial income gap also shows the trend of decreasing first and then expanding. (4) Further research finds that although the level of factor misallocation between provinces in China is significantly lower than the average level of factor misallocation between the three major industries within each province, it has a stronger explanatory power on the income gap between provinces. Correcting the total factor misallocation and labor misallocation between provinces can significantly narrow the income gap between provinces in China, and correcting the total factor misallocation and labor misallocation between provinces in 2019 can reduce the income gap between provinces by 51.48% and 81.68%, respectively. Correcting the inter-provincial capital misallocation alone will widen the income gap between provinces, and correcting the inter-provincial capital misallocation in 2019 will increase the income gap between provinces by 112.21%.

The research conclusion of this article indicates that there is a serious factor misallocation among the three major industries in various provinces of China, and the factor misallocation has a strong explanatory power for the income gap. It also indicates that part of the current income gap in China can be attributed to differences in factor allocation efficiency. This article explains the reasons for the widening income gap from the perspective of factor allocation, and takes a meaningful step towards the mechanism by which factor misallocation affects economic growth. However, this article did not further delve into the linkage effect and transmission mechanism among factor misallocation, income distribution gap, consumption demand and economic growth. This will constitute an important research direction for the author in the future. In the future, we will further systematically examine how factor misallocation leads to changes in income distribution and consumption structure, thereby affecting economic growth. From the perspective of factor allocation, we will search for the root causes of fluctuations in income distribution and consumption demand, Propose more effective countermeasures to reduce China’s income gap and expand domestic demand.

Based on the above main research conclusions, this paper puts forward the following countermeasures from the perspective of factor flow:

The first is to accelerate the level of agricultural mechanization, digitalization and modernization, increase financial support for agricultural production technology transformation, guide social capital to tilt to the agricultural field, and improve the in-tensity of agricultural capital. It is recommended to further improve the financial policies and regulations to support and benefit agriculture, encourage and guide state-owned banks, joint-stock banks and urban commercial banks to increase support for agricultural production technology mechanization and digital transformation projects, and give play to the function of "financial hematopoiesis" to alleviate the dilemma of insufficient input of agricultural capital factors.

The second is to strive to resolve the contradiction between the future employment direction of the tertiary industry and the current shortage of labor allocation. The vigorous development of the digital economy has led to the continuous acceleration of the digital transformation of the service industry, and the tertiary industry will remain a prominent growth point of China’s economy and an important industrial field for providing jobs in the future. According to the calculations in this paper, the tertiary industry in various provinces generally has the dilemma of insufficient input of labor factors. Therefore, it is recommended to vigorously develop the service industry in the tertiary industry and improve the absorption capacity of the tertiary industry for labor and employment. In addition, it is necessary to give full play to the social division of work of Consumer Internet Enterprises [41], and guide the transfer of low-skilled redundant labor in agriculture and rural areas to low-skilled industrial sectors within the tertiary industry, so as to achieve more effective social division of labor results and factor allocation efficiency.

The third is to pay attention to the rational flow allocation of capital factors as well while correcting the misallocation of labor as the main direction of attack, in order to give play to the superposition effect of the optimal allocation of multiple factors, improve the total factor productivity of the economy, and further optimize the income distribution pattern. This paper shows that only correcting capital misallocation or only correcting labor misallocation has a significantly weaker effect on narrowing income gap within industries, between industries and between regions than that of correcting total misallocation. Therefore, although the factor misallocation between the three major industries in China’s provinces is mainly caused by labor misallocation, the importance of correcting capital misallocation should not be ignored in the process of correcting factor misallocation and optimizing income distribution pattern.

The fourth is to promote the free flow and allocation of production factors in a larger spatial range by accelerating the construction of a unified domestic market and accelerating the integration of factor markets, in order to effectively narrow the income gap between regions. The research conclusion of this paper shows that the regional integration and factor allocation improvement within each province can no longer narrow the income gap between provinces and industries in China, and cannot solve the main contradiction of China’s current imbalance and insufficient development. Therefore, it is necessary to further accelerate the coordinated development of the Beijing-Tianjin-Hebei region, the construction of the Guangdong-Hong Kong-Macao Greater Bay Area, and the integrated development of the Yangtze River Delta, and promote the breaking of provincial administrative boundaries of production factors and carry out free flow and efficient allocation in a broader space by accelerating the construction of a unified domestic market, so as to correct the distortion of production factor allocation, accelerate the creation of a total factor productivity-driven economic growth model and achieve a fair and just income distribution.
